# In Vitro Characterization of the Immune Response to an Epitope Ensemble Vaccine Against Rhinovirus in Pediatric Asthma and Adults With Chronic Obstructive Pulmonary Disease: Protocol for an Observational and Exploratory Study

**DOI:** 10.2196/73383

**Published:** 2025-06-30

**Authors:** Sara Alonso Fernandez, Raquel Reyes-Manzanas, Susana Camara, Juan Mozas-Gutierrez, Myriam Calle-Rubio, Juan Rodriguez-Hermosa, Andres Bodas-Pinedo, Santiago Rueda Esteban, Esther M Lafuente, Jesús Reiné, Pedro A Reche

**Affiliations:** 1 Department of Immunology, Ophthalmology and ORL Faculty of Medicine Universidad Complutense de Madrid Madrid Spain; 2 Department of Paediatrics, Oxford Vaccine Group University of Oxford Oxford United Kingdom; 3 Unit of Pulmonology Hospital Clínico San Carlos Madrid Spain; 4 Department of Medicine School of Medicine Universidad Complutense de Madrid Madrid Spain; 5 Health Research Institute (IdISSC) Hospital Clínico San Carlos Madrid Spain; 6 Paediatrics Unit Hospital Clínico San Carlos Madrid Spain; 7 Department of Clinical Sciences Liverpool School of Tropical Medicine Liverpool United Kingdom; 8 Centre of Health Studies Universidad del Valle de Guatemala Guatemala City Guatemala

**Keywords:** asthma, COPD, chronic obstructive pulmonary disease rhinovirus, epitopes, vaccine, protocol, study design, exploratory, observational study.

## Abstract

**Background:**

Human rhinoviruses (HRVs) are the leading cause of upper respiratory tract infections, responsible for over half of all such infections. Infection rates among young children can reach as high as 8-12 episodes per year. While HRV infections typically result in mild common colds, they can also lead to more severe respiratory conditions, often in conjunction with bacterial coinfections. In addition, HRVs are implicated in the exacerbation of obstructive respiratory diseases, including asthma and chronic obstructive pulmonary disease (COPD). T-cell responses play a crucial role in the immune defense against HRV. However, in patients with obstructive respiratory diseases, altered or dysregulated T-cell responses to HRV may not only fail to efficiently eliminate the virus but can also exacerbate inflammation and airway remodeling. Therefore, a deeper understanding of T-cell–mediated responses in the context of HRV infection, especially in vulnerable populations like those with COPD, is critical. It can provide new insights into mechanisms of both protection and disease exacerbation, potentially guiding the development of targeted therapies or vaccines that enhance protective immunity while minimizing harmful inflammation.

**Objective:**

This study aims to (1) determine the population-wide coverage of HRV-specific T-cell responses, (2) characterize HRV-specific T-cell recall responses in disease cohorts compared to age-match healthy controls, and (3) identify biomarkers of protection and susceptibility within disease cohorts through a comparative analysis.

**Methods:**

Participants with asthma and those with COPD, aged 5-15 and 40-70 years, respectively, will be recruited alongside healthy age-matched controls. Peripheral blood samples will be collected following informed consent from adult participants and from parents or guardians of minors, as applicable. Clinical, demographic, immunological, and genetic responses will be assessed both prior to and following in vitro stimulation with a pool of HRV-specific T-cell epitopes. Flow cytometry and functional assays will be used to analyze T-cell responses to HRV epitopes in the context of obstructive respiratory diseases.

**Results:**

This study was funded in January 2023 by the Ministry of Science and Innovation of Spain. The primary aim of the study was achieved within the same year. Recruitment for the secondary and tertiary aims is currently ongoing. Preliminary findings highlight the potential significance of HRV-specific T-cell responses in individuals with asthma and those with COPD. A detailed characterization of these immune responses will provide critical insights into host-pathogen interactions and may serve as a foundation for the development of effective T-cell–based vaccines or immunotherapies targeting HRV.

**Conclusions:**

Here, we present an ethically approved study protocol for an observational and exploratory study investigating a novel epitope-based vaccine targeting HRV, with a focus on pediatric asthma and adult COPD cohort populations.

**International Registered Report Identifier (IRRID):**

DERR1-10.2196/73383

## Introduction

### Rhinovirus and Its Context in Obstructive Respiratory Disorders

Human rhinoviruses (HRVs) are responsible for over half of all upper respiratory tract infections (URIs), with infection rates in young children reaching as high as 8-12 episodes per year [1]. HRV infections typically cause mild symptoms such as rhinorrhea, sore throat, coughing, sneezing, nasal congestion, and malaise [1]. They can also lead to more severe conditions, including acute otitis media and rhinosinusitis, often in association with bacterial coinfections [1,2]. In addition, HRVs are implicated in the development of lower respiratory tract diseases, such as pneumonia, bronchitis, and bronchiolitis [1], as well as in the exacerbation of asthma [3] and chronic obstructive pulmonary disease (COPD) [4].

The susceptibility to severe HRV infections and reinfections is influenced by a range of environmental and genetic factors, including impaired interferon responses and pre-existing conditions namely asthma and COPD [5]. Given the high frequency of HRV infections and their broad clinical implications, effective therapeutic and preventive strategies are urgently needed, as current treatments primarily focus on symptom relief, with no vaccine available to date. The development of such strategies would have significant public health benefits.

### HRV Serotypes and Antigens

**Figure 1:**
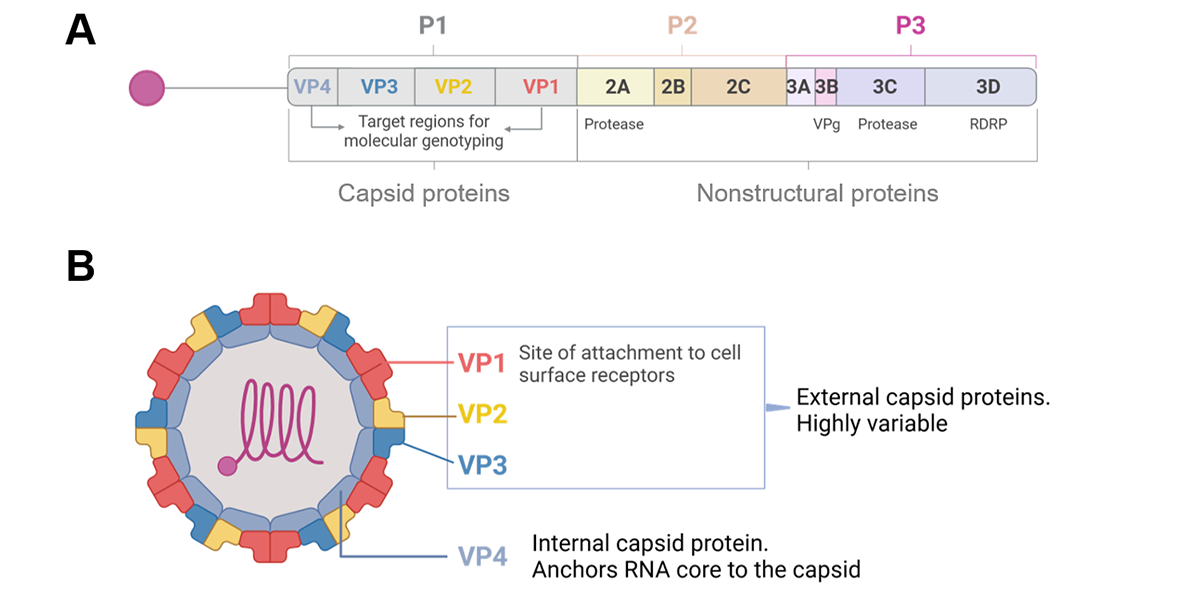
Human rhinovirus proteome and structure. (A) Capsid and nonstructural proteins. (B) Rhinovirus capsid Viral Proteins (VP) related to the site of attachment to cell surface receptors (VP1-3) and internal capsid protein (VP4). Figure created with Biorender. RDRP: RNA-dependent RNA polymerase.

HRV is a small, nonenveloped virus belonging to the Picornaviridae family and the *Enterovirus* genus. It has a positive-strand RNA genome of approximately 7.2 kb [1,2]. The viral genome is translated into a single polyprotein, which undergoes proteolytic processing to generate 11 distinct viral proteins (Figure 1A). The HRV capsid is composed of 4 viral proteins (VPs): VP1, VP2, VP3, and VP4 (Figure 1B). With the exception of VPg, the remaining viral proteins are nonstructural and are involved in viral replication and assembly (Figure 1A). Notably, the capsid proteins exhibit considerable heterogeneity, leading to a broad range of antigenic diversity [6], particularly in VP1, which mediates viral entry by interacting with cell surface receptors (Figure 1B) [7].

HRV demonstrates exceptional diversity, with 3 recognized species: HRV-A, HRV-B, and HRV-C.

Each species includes numerous distinct serotypes, which can be differentiated through serological methods. At present, there are approximately 83 HRV-A, 32 HRV-B, and 55 HRV-C serotypes in circulation. Furthermore, high-resolution sequencing has identified multiple HRV serotypes within each serotype. HRV-A and HRV-C are primarily responsible for the majority of HRV-related respiratory infections and are more prevalent than HRV-B in individuals with asthma or COPD exacerbations [8-10]. Notably, HRV-C is associated with more severe infections compared to HRV-A and HRV-B species [11]. Given the significant impact of HRV on human disease, the development of an effective rhinovirus vaccine must target primarily HRV-A and HRV-C serotypes.

### Rhinovirus Infection and Immune Response

**Figure 2:**
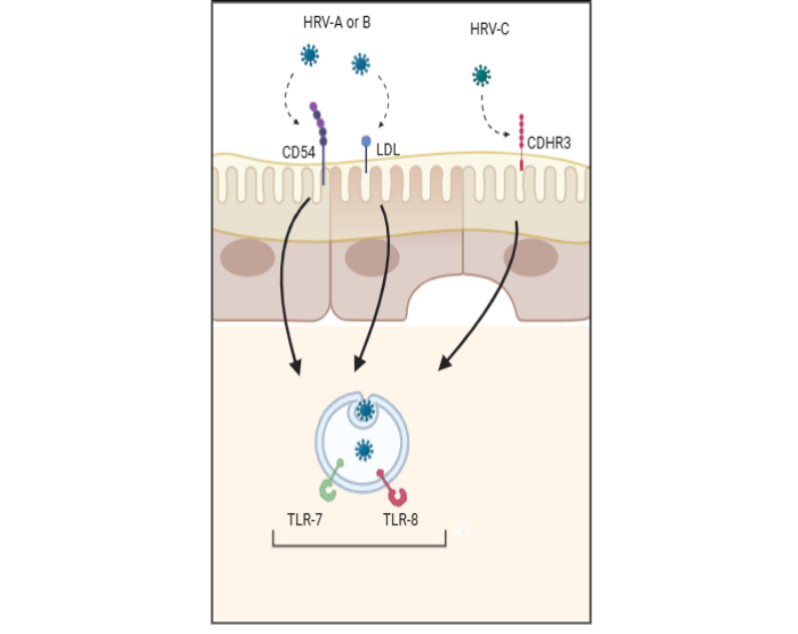
Human rhinovirus entry into the respiratory epithelium. Schematic illustrating human rhinovirus serotypes A, B, and C and their respective viral entry receptors. HRV-A and B primarily bind to the ICAM-1 (CD54) or LDL receptor, whereas HRV-C typically targets the CDHR3 receptor. Upon endosomal entry, TLR receptors 7 and 8 recognize the virus, triggering the release of IFN-γ and pro-inflammatory cytokines. Figure created with Biorender. CD54: cluster of differentiation 54; CDH3: Cadherin-Related Family Member 3; HRV: human rhinoviruses; ICAM-1: intercellular adhesion molecule 1; LDL: low-density lipoprotein; TLR: toll-like receptor.

HRV primarily infects epithelial cells of the lower respiratory tract, although it can also affect the upper respiratory tract (Figure 2). The viral entry process begins when the VP1 capsid protein attaches to various cell surface receptors. Most HRV-A and HRV-B serotypes enter airway epithelial cells via the ICAM-1 (intercellular adhesion molecule 1) receptor (also known as CD54), while a smaller fraction uses the low-density lipoprotein receptor [11]. In contrast, HRV-C serotypes predominantly use the cadherin-related family member 3 as their primary receptor for viral entry [12]. Once inside the host cell, the HRV genome is detected by endosomal toll-like receptors (TLRs) such as TLR7 and TLR8, triggering the production of type I interferons and proinflammatory cytokines such as RANTES, IP-10, interleukin (IL)-6, IL-8, and C-X-C motif chemokine ligand 5. The levels of these cytokines may influence the pathogenesis of symptomatic HRV infections, as they play a key role in initiating adaptive immune responses [2].

In response to HRV infection, affected individuals develop serotype-specific neutralizing immunoglobin G antibodies in their serum, as well as secretory immunoglobin A antibodies in the airways. However, these neutralizing antibodies target the highly variable, surface-exposed regions of the VP1, VP2, and VP3 capsid proteins, which are serotype-specific. Consequently, there is minimal or no cross-neutralization activity between different HRV serotypes [13]. While these antibodies do not appear to contribute significantly to the pathogenesis of HRV infections [1], their lack of cross-neutralization presents a substantial challenge for vaccine development.

Despite the critical role of T cells in immunity, the study of T-cell responses to HRV has been relatively neglected in favor of antibody research. This discrepancy is largely due to methodological limitations, as antibodies are relatively easy to measure, while antigen-specific T-cell responses require more complex cellular assays. Nonetheless, there is compelling evidence of the importance of HRV-specific T cells, which play a crucial role in initiating both cellular and humoral immune responses [14]. Circulating memory CD4^+^ and CD8^+^ T cells capable of recognizing HRV-specific antigens have been identified in healthy individuals [15]. HRV-specific CD4^+^ T cells typically adopt a T helper type 1 phenotype, producing proinflammatory cytokines such as interferon-gamma, IL-2, tumor necrosis factor-alpha, and IL-6. These T helper type 1 cells, in turn, promote the activation and differentiation of HRV-specific cytotoxic CD8^+^ T cells, which are essential for clearing HRV infections through their ability to recognize and destroy infected cells [16].

Unlike B cells, which can only recognize accessible antigens, T cells can target both exposed and hidden antigenic components. They recognize small peptide fragments of antigens displayed on the surface of antigen-presenting or infected cells, bound to major histocompatibility complex molecules. In humans, major histocompatibility complex molecules are known as human leukocyte antigen (HLA) molecules. Specifically, CD8^+^ T cells recognize antigens presented by HLA class I (HLA I) molecules, while CD4^+^ T cells interact with antigens presented by HLA class II (HLA II) molecules [17]. In contrast to the serotype-specific nature of HRV antibodies, T cells have been shown to respond to multiple HRV serotypes, suggesting the presence of conserved epitopes shared across different HRV serotypes [18,19].

### Rationale of the Study

Asthma and COPD are prevalent respiratory conditions that significantly affect public health, leading to increased morbidity, mortality, and health care costs. One of the major triggers of exacerbations in these diseases is infection with the HRV, which is responsible for a substantial number of acute episodes. These viral infections exacerbate the symptoms of asthma and COPD, increasing airflow obstruction, inflammation, and the odds of hospitalization.

The development of a vaccine targeting HRV has the potential to greatly reduce viral-induced exacerbations, improving the quality of life and health outcomes for individuals with asthma and those with COPD. An epitope-based vaccine consisting of conserved viral epitopes offers the possibility of inducing protective immunity against all HRV serotypes.

The final aim of this proposal is to advance the development of an epitope-based vaccine against HRV. To that end, we will explore the role of HRV-specific T-cell immunity in individuals with COPD and asthma, since these two groups will be the main beneficiaries of a potential vaccine. Furthermore, understanding how T-cell responses to HRV differ in these populations could provide valuable insights into the pathogenesis of viral-induced exacerbations and guide the design of future immunotherapies.

## Methods

### Study Aims

There are primary and exploratory outcomes. The primary outcome of the study is determining population-wide coverage of HRV-specific T-cell responses based on HLA binding profile and high throughput analysis of T-cell recall responses. This aim will help us to determine the responses in the population to viral epitopes across diverse HLA backgrounds, which is a critical step for the rational design of broadly effective T-cell–based vaccines. The exploratory aims of the study are the characterization of HRV-specific T-cell recall responses in the context of obstructive respiratory disorders in comparison to age-matched healthy controls. This exploratory aim will elucidate disease-associated alterations in T-cell immunity and reveal whether impaired or dysregulated T-cell responses contribute to increase disease severity or susceptibility to HRV-induced exacerbations. This aim includes profiling cytokine production, T-cell subset distribution, and activation or exhaustion markers. Such biomarkers will be useful for risk stratification for targeted immunomodulatory strategies. In addition, we will also explore comparative T-cell responses elicited by in vitro immunization of HRV epitopes in healthy and patient individuals. This aim will be instrumental for in vitro validation of vaccines aimed at vulnerable populations.

### Study Setting and Recruitment

All study visits and associated procedures for both pediatric and adult cohorts will be conducted at the Hospital Clínico San Carlos, located in Madrid, Spain. During the study visit, participants will undergo a series of assessments and blood sample collections, all of which will be carried out by trained medical and research personnel in accordance with standard clinical protocols.

All participants will be enrolled in the study to attend a single visit to the hospital, coinciding with their routine monthly clinical check-up. This visit will serve as the primary data collection point for both pediatric and adult cohorts. Prior to any sample collection, participants (or their legal guardians, in the case of pediatric participants) will be required to review and sign a consent form, ensuring informed consent is obtained. During the visit, comprehensive clinical data will be gathered. In addition, participants will provide biological samples for immunological analysis, including serum and peripheral blood mononuclear cells (PBMCs), as well as blood samples for transcriptomic analysis.

### Eligibility Criteria

The inclusion and exclusion criteria for children with asthma and adult participants with COPD enrolled in this study are outlined in Textboxes 1 and 2.

**Inclusion and exclusion criteria for participants with pediatric asthma.**

**Inclusion criteria**

- Age between 5 and 15 y with a paired sex distribution (ratio 1:1), equivalent age, and BMI.
- Participant who speaks Spanish fluently to ensure a comprehensive understanding of the research project and proposed participation.
- Ability to provide informed consent both individually and parental (parents or legal guardian).
- Awaiting a minor procedure in the case of healthy participants.
- Complete diagnosis and clinical score compatible with the respiratory disorders under study. The asthma diagnosis must have been identified at least 6 mo prior, with well-controlled asthma by a clinical team experienced and familiar with respiratory diseases. Compatible asthma will present the following symptoms: wheezing (current, persistent, or triggered), cough (including nocturnal), shortness of breath, nocturnal symptoms, and diurnal and seasonal variations.
- Asthma positivity thresholds in minors: (1) obstructive spirometry: forced expiratory volume/forced vital capacity (FEV/FVC) ratio less than 70%. Consider forced expiratory volume in 1 second/forced vital capacity (FEV1/FVC) with a ratio of less than 80% (or below the lower limit of normal if available) as a positive test for detecting obstructive airway disease (obstructive spirometry). (2) FeNO (fractional exhaled nitric oxide): 35 ppb or higher (3) Bronchodilator reversibility: Improvement in FEV1 of 12% or more. Consider a bronchodilator reversibility test in children and adolescents (5-16 y of age) with obstructive spirometry (FEV1/FVC ratio less than 70%). Consider an improvement in FEV1 of 12% or more as a positive test. (4) Peak flow variability: More than 20%.
- BMI: healthy weight (BMI above the 2^nd^ percentile and below the 85^th^ percentile).

**Exclusion criteria**

- Inability to provide individual or parental consent. Inability to comprehend reading, visual, or cognitive information that would prevent consent.
- Does not speak or understand Spanish.
- Children under 5 y old or adults.
- Immunocompromised minors or any other disease that leads to altered immunity.
- Inappropriate BMI: (1) Overweight: BMI above the 85^th^ percentile and below the 95^th^ percentile. (2) Obesity: BMI equals or above the 95^th^ percentile. (3) Underweight: BMI below the 2nd percentile.
- Taking medications that may affect the immune system, such as oral steroids, nasal steroid sprays, antibiotics, and disease-modifying antirheumatic drugs.
- Any acute illness – unexplained symptoms in the last 14 d.
- Have received antibiotics, oral steroids, or nasal steroid sprays in the last 28 d.
- More than one asthma exacerbation (as defined by the American Thoracic Society) in the last 12 mo.
- Taking anticoagulants.
- Participating simultaneously in another clinical trial unless it is observational or in a follow-up phase (noninterventional).
- History of significant cardiopulmonary disease (excluding stable hypertension and asthma in treatment steps 2 and 3), or diseases associated with altered immunity, including diabetes, alcohol abuse, malignancies, and rheumatological conditions.
- Pregnancy.
- Taking any medication except those on the “allowed list” (statins, antihypertensives, antidepressants, bisphosphonates, hormone replacement therapy, vitamin supplements, antacids, nicotine replacement therapy, inhaled steroids up to 800 mg equivalent of Beclomethasone dipropinate, inhaled beta-2 agonists, and leukotriene receptor antagonists).
- Not presenting any clinical respiratory condition due to bacterial infection (*Streptococcus pneumoniae*) or viral infection (RSV, SARS-CoV-2, etc) or coinfections requiring hospitalization, and that have not occurred within at least the past month.

**Inclusion and exclusion criteria for adult chronic obstructive pulmonary disease participants.**

**Inclusion criteria**

- Age between 35 and 75 y with a paired sex distribution (ratio 1:1), equivalent age, and BMI.
- Participant who speaks Spanish fluently to ensure a comprehensive understanding of the research project and proposed participation.
- Ability to provide individual informed consent.
- Awaiting a minor procedure in the case of healthy participants.
- Complete diagnosis and clinical score compatible with the respiratory disorders under study. The chronic obstructive pulmonary disease (COPD) diagnosis must have been identified at least 6 mo prior by a clinical team experienced and familiar with respiratory diseases.
- Smokers or ex-smokers (of at least 10 pack-years).
- COPD positivity thresholds: (1) COPD diagnosis defined by spirometry as forced expiratory volume in 1 second/forced vital capacity (FEV1/FVC) ratio <0.7 (postbronchodilator). (2) Consider FEV1/FVC with a ratio of less than 70% (or below the lower limit of normal if this value is available) as a positive test for detecting obstructive airway disease (obstructive spirometry).
- BMI: Healthy weight (BMI above the 2^nd^ percentile and below the 85^th^ percentile).

**Exclusion criteria**

- Inability to provide individual or parental consent. Inability to understand reading, visual, or cognitive information that would prevent consent.
- Does not speak or understand Spanish.
- History of or current drug or alcohol abuse (frequent alcohol consumption: men and women should not regularly drink >3-4 units/d and > 2-3 units/d, respectively).
- Immunocompromised patients or any other disease that leads to altered immunity.
- Inadequate BMI: (1) Overweight: BMI above the 85^th^ percentile and below the 95^th^ percentile. (2) Obesity: BMI equals or above the 95^th^ percentile. (3) Underweight: BMI below the 2^nd^ percentile.
- Taking medications that may affect the immune system. For example, oral steroids, nasal steroid sprays, antibiotics, and disease-modifying antirheumatic drugs.
- Any acute illness – unexplained symptoms in the last 14 d.
- Have received antibiotics, oral steroids, or nasal steroid sprays in the last 28 d.
- Taking anticoagulants.
- Participating simultaneously in another clinical trial unless it is observational or in a follow-up phase (noninterventional).
- History of significant cardiopulmonary disease (excluding stable hypertension and asthma in treatment steps 2 and 3), or diseases associated with altered immunity, including diabetes, alcohol abuse, malignancies, and rheumatological conditions.
- Pregnancy.
- Taking any medication except those on the “allowed list” (statins, antihypertensives, antidepressants, bisphosphonates, hormone replacement therapy, vitamin supplements, antacids, nicotine replacement therapy, inhaled steroids up to 800 micrograms equivalent of BDP, inhaled beta-2 agonists, and leukotriene receptor antagonists).
- Not presenting any clinical respiratory condition due to bacterial infection (*Streptococcus pneumoniae*) or viral infection (RSV, SARS-CoV-2, etc) or coinfections requiring hospitalization, and that have not occurred within at least the past month.
- Positive lateral flow test for SARS-CoV-2.
- Acute COPD exacerbation in the last 3 mo or more than 3 exacerbations in the last 12 mo requiring oral steroidsor hospitalization.
- On ambulatory or long-term oxygen therapy.
- If there is evidence of severe obstruction (FEV1 <50% of postbronchodilator predicted), participants will be excluded from the study.

### Study Timeline and Duration

This project is planned for 3 years (2023-2026) where the major aims are the following: (1) determination of the population-wide coverage of HRV-specific T-cell responses (2023, first year), (2) characterization of HRV-specific T-cell recall responses in diseases cohorts compared to age-match healthy controls (2024-2026), and (3) comparative analysis and identification of biomarkers of protection and susceptibility within disease cohorts (2025-2026).

### Sample Size Calculation

For this exploratory study, the aim is to compare the in vitro immune response between children with asthma or adults with COPD and those without, based on blood samples. Given the exploratory nature of the study and the expected parametric distribution of the population, a sample size of 40 participants (20 per group) for each study (asthma and COPD) will be used. The sample size was estimated to detect ~15% differences in HRV-specific T-cell responses between healthy and disease participants at an α level of .05 (2-tailed) and a β value of .2. This sample size is intended to provide preliminary insights into HRV-specific immune responses, serving as a foundational step toward larger, more comprehensive research studies. While the limited number of participants requires cautious interpretation of results, the primary objective is to identify immunological trends and generate testable hypotheses.

### Ethical Considerations

This exploratory study protocol has been approved by the Ethical Board Committee of the Hospital Clínico San Carlos (23/799-E). The study will be conducted in accordance with the Declaration of Helsinki and Good Clinical Practice (GCP) guidelines. All participants provided their written informed consent to participate in this study. Participant confidentiality will be maintained throughout the study. All data will be anonymized during processing and stored securely.

## Results

### Epitope Selection

**Figure 3:**
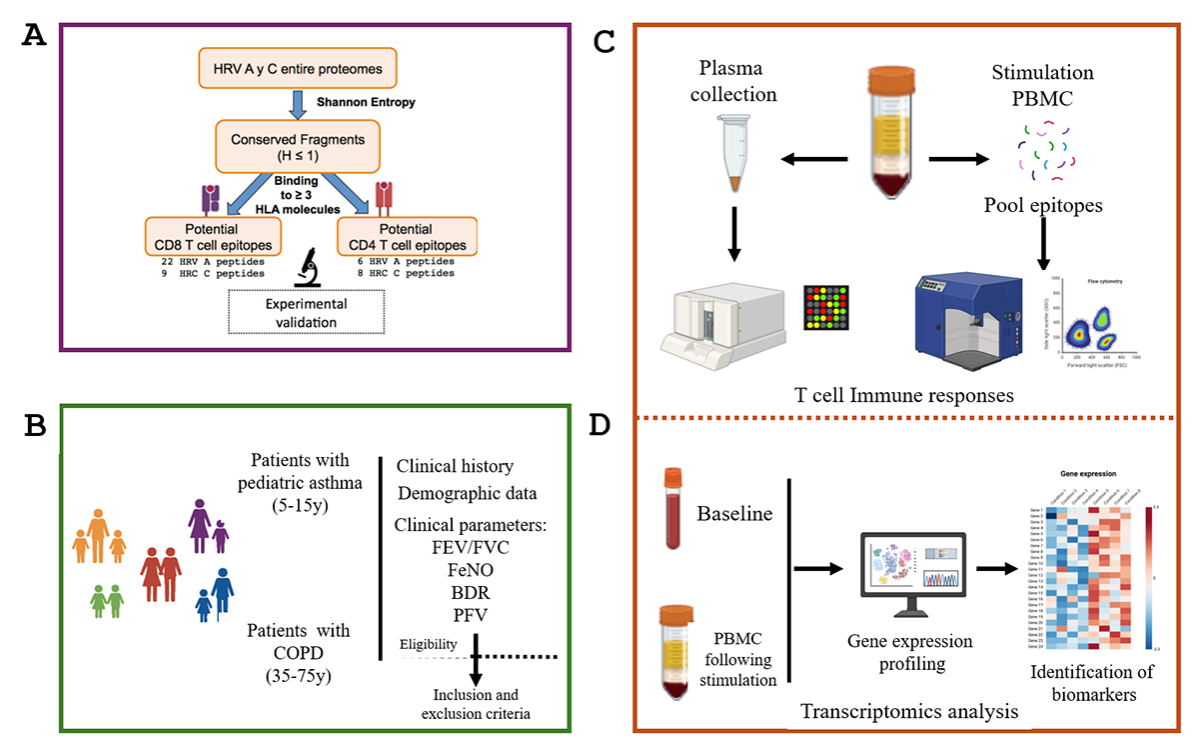
Human rhinovirus epitope selection and analysis overview. (A) Computer-aided selection of epitopes. HRV sequence variability was evaluated via Shannon entropy, and peptides were selected based on HLA-I and HLA-II binding affinity using RANKPEP. Epitope peptides were synthesized and validated experimentally. (B) Study design and participant recruitment. Pediatric and adult patients were selected according to predefined inclusion and exclusion criteria. (C) T-cell–specific immune responses will be assessed in vitro through stimulation with epitope pools, followed by flow cytometry and Luminex assays. (D) Transcriptional analysis of blood samples (both unstimulated PBMCs and following epitope stimulation, PBMCs) will be conducted to identify gene expression profiles associated with epitope protective immunity. Figure created with Biorender. BDR: bronchodilator response; CD4: cluster of differentiation 4; CD8: cluster of differentiation 8; CDHR3: cadherin-related family member 3; COPD: chronic obstructive pulmonary disease; FeNO: fractional exhaled nitric oxide; FEV: forced expiratory volume; FEV/FVC: ratio of forced expiratory volume in 1 second to forced vital capacity; FVC: forced vital capacity; HLA: human leukocyte antigen; HRV: human rhinovirus; ICAM-1: intercellular adhesion molecule 1; LDL receptor: low-density lipoprotein receptor; PBMC: peripheral blood mononuclear cells; PFV: peak flow variability.

HRVs are characterized by their great diversity, circulating in nature over 150 antigenically distinct serotypes of HRV. We identified HRV-A and C-specific T-cell epitopes from a reduced set of potential candidates using a computer-assisted strategy (Figure 3). Shannon Entropy was used to assess HRV sequence variability, isolating nonvariable regions for T-cell epitope prediction. Peptides were selected based on predicted binding to at least 3 distinct HLA I or HLA II molecules, using RANKPEP software developed by Reche et al [20]. The immunogenicity of 37 peptides was tested in interferon-gamma ELISPOT recall assays with PBMCs from 14 HLA-typed participants; 25 peptides were positive in at least one participant. Further validation assays led to the first reported HRV-specific CD8^+^ T-cell epitopes (5 epitopes from HRV-A and 4 from HRV-C) [21]. In addition, 7 HRV-specific CD4^+^ T-cell epitopes were identified (3 from HRV-A and 4 from HRV-C) [22]. Epitopes will be generated by chemical synthesis, dissolved in dimethyl sulfoxide and combined into pools. Three epitopes’ pools will be assembled: HRV CD4^+^ pool, HRV CD8^+^ pool, and HRV pool (including all the epitopes; Figure 3A).

### Clinical Measurements

Comprehensive and demographic medical data will be collected, including exacerbation history, GOLD (Global Initiative for Chronic Obstructive Lung Disease) stage (for COPD), spirometry (forced expiratory volume in 1 second), and current treatment regimen (Figure 3B). This information will be used to correlate immune responses to HRV-specific T-cell epitopes with key demographic and clinical variables such as age, gender, disease severity, and medication, thus enabling a more holistic view of the immune landscape in obstructive respiratory diseases that will provide insights into HRV-related exacerbations across different demographics and clinical parameters between cohorts.

### Immune Measurements

Systemic T-cell antigen–specific immune responses will be measured in vitro following exposure to HRV peptide pools (Figure 3C). These responses will be assessed to understand the relationship between immune reactivity and clinical parameters in individuals with asthma and those with COPD. Blood samples, including plasma and PBMCs, will be collected from participants for this analysis. Statistical comparisons of T-cell responses with HRV in disease and healthy participants will be carried out, taking into consideration clinical parameters, sex, age, and potential confounding factors such as medication.

### Genetic Measurements

Whole blood samples will be collected from participants at baseline (single visit) to establish a comprehensive profile of individuals with asthma and those with COPD (Figure 3D). In addition, PBMCs isolated from the same participants following stimulation in vitro with HRV peptide pools will be used for RNA sequencing to assess gene expression and regulation in response to HRV-specific stimulation (Figure 3D). This analysis will elucidate key insights into the immune mechanism driving HRV-related disease exacerbations and identify biomarkers that could predict clinical outcomes.

Collectively, from 2023 up to 2025 we have accomplished the primary outcome of the study, and we have recently completed the recruitment of the adult cohort of participants diagnosed with COPD, and their respective age-matched controls. We expect to start the recruitment of the pediatric asthma population cohort during this year and complete the T-cell responses data analysis, from both population cohorts, according to our study timeline.

## Discussion

### Toward a T-Cell–Based Vaccine Against HRV

HRV is a major contributor to respiratory infections and exacerbations in individuals with obstructive respiratory disorders. Despite the widespread impact of HRV, no licensed vaccine currently exists. This observational and exploratory study proposes an innovative advance in the development of an epitope-based vaccine against HRV in individuals with COPD and asthma. Both vulnerable groups will be the main beneficiaries of a potential vaccine. We hypothesized that a peptide pool consisting of HRV-A and C T-cell epitopes could serve to develop an effective HRV vaccine against all A and C serotypes. This study will allow the exploration and the assessment of systemic T-cell immune responses against a specific pool of selected HRV epitopes, previously identified in silico, and experimentally validated by flow cytometry in vitro [21,22].

HRV-specific CD8+ T-cell epitopes include 8 typical 9-mer peptides and an unusually long 16-mer peptide restricted by HLA-A*02:01 [21], which also induced CD4⁺ T-cell responses [22]. These CD8^+^ T-cell epitopes induced functional cytotoxic CD8⁺ T-cell responses and could potentially cover up to 87% of the population. The selected HRV-specific CD4^+^ T-cell epitopes consist of 7 peptides characterized for their promiscuous binding to HLA class II molecules and could potentially be recognized by over 95% of the global population [22]. Collectively, these HRV-specific T-cell epitopes provide a strong foundation for future HRV vaccine development. Other studies also support the potential use of HRV-specific T-cell epitopes for vaccine development.

A previous study in a mouse model identified dominant CD4^+^ T-cell epitopes in HRV type 1A that were distinct from antibody recognition sites. These epitopes elicited strong proliferative responses and exhibited broad cross-reactivity across HRV serotypes, suggesting their potential inclusion in broadly protective T-cell–based vaccines [19]. In addition, a recent study designed a multiepitope vaccine in silico, targeting conserved regions only on HRV-C, demonstrating strong antigenicity, stability, and solubility [23] with validation via C-ImmSim simulations [24].

Our methodological approach will examine HRV-specific T-cell immunity using peripheral blood samples from individuals diagnosed with obstructive respiratory diseases such as asthma and COPD and correlate those immune responses to clinical parameters and transcriptional signatures. This multiparametric combined analysis will facilitate the potential identification of protectionor susceptibility biomarkers or signatures within those vulnerable population cohorts before and following stimulation with the HRV epitopes. We anticipate that we will be able to identify altered HRV-specific T-cell responses in patients with asthma and those with COPD compared to healthy controls. Presumably, we will also find that factors such as age, disease severity, and medication may influence these responses, potentially revealing biomarkers of susceptibility or protection. Clearly, understanding T-cell responses to HRV within these clinical contexts is key for vaccine development. Furthermore, the study could also provide significant insights into the underlying mechanisms of viral-induced exacerbations as well as guidelines for the development of future immunotherapies.

### Strengths and Limitations

This exploratory study will allow the identification of immunological trends and potential biomarkers related to HRV-specific T-cell responses in asthma and COPD, helping to fill a critical gap in current respiratory immunology. The integration of detailed clinical and demographic data such as age, gender, disease severity, and medication use will add translational relevance and may uncover factors influencing susceptibility or protection. However, this study has also limitations. The small sample size reduces statistical power and limits the generalizability of the results. Furthermore, this exploratory study may not capture longitudinal changes in the immune responses, which are important to characterize disease progression. Furthermore, potential confounding factors such as varying treatment regimens and comorbidities may hinder potential immune signatures connected to clinical outcomes. Despite these limitations, the study is well-positioned to provide valuable preliminary insights and inform the design of larger more definitive investigations.

### Future Directions

The outcomes from this exploratory study will establish a foundation for larger-scale studies including those on mucosal immune responses following stimulation and their correlation to systemic and lung responses. Future studies will focus on expanding the sample size and incorporating both peripheral blood and mucosal samples, alongside longitudinal analyses to evaluate how HRV-specific T-cell responses change over time and correlate with disease progression. Future research will also tackle more diverse and clinically relevant cohorts, including different asthma endotypes and COPD phenotypes across multiple settings. Furthermore, it will deepen the characterization of T-cell subsets and their interaction with other immune cells, using advanced techniques such as spectral flow cytometry, and incorporate multiomics approaches like transcriptomics and proteomics. All together will enable a system-level view of immune responses, enhance our understanding of HRV-driven immunopathology in obstructive airway diseases and facilitate the discovery of novel biomarkers and therapeutic targets.

## Abbreviations

COPD: chronic obstructive pulmonary disease
FEV: forced expiratory volume
GCP: Good Clinical Practice
GOLD: Global Initiative for Chronic Obstructive Lung Disease
HLA: human leukocyte antigen
HRV: human rhinovirus
IL: interleukin
PBMC: peripheral blood mononuclear cell
TLR: toll-like receptor
URI: upper respiratory tract infection
VP: viral protein
